# A Comparative Study between Vitrectomy with Internal Tamponade and a New Modified Fiber Optic Illuminated Ando Plombe for Cases of Macular Hole Retinal Detachment in Myopic Eyes

**DOI:** 10.1155/2015/841925

**Published:** 2015-10-15

**Authors:** Ahmed M. Bedda, Ahmed M. Abdel Hadi, Muhammad S. Abd Al Shafy

**Affiliations:** ^1^Ophthalmology Department, Faculty of Medicine, Alexandria University, Alexandria 21526, Egypt; ^2^General Ophthalmology Hospital of Alexandria, Alexandria 21547, Egypt

## Abstract

*Aim.* To compare pars plana vitrectomy (PPV) with silicone tamponade or gas (Groups Ia and Ib) and a new modified Ando plombe equipped with a fiber optic light (Group II) for cases with macular hole retinal detachment (MHRD) in high myopic eyes (axial length > 26 mm). *Methods.* A prospective interventional randomized case series included 60 eyes (20 in each group). Successful outcome was considered if the retina was completely attached at the end of the follow-up period. Complications were identified for each group. *Results.* Visual acuity improved by 37.31%, 40.67%, and 49.40% in Groups Ia, Ib, and II, respectively. The success rate was 55%, 60%, and 100% in Groups Ia , Ib, and II, respectively, with a statistically significant difference between Groups Ia, Ib, and II (*p* < 0.001 in Ia, *p*: 0.002 in Ib). Complications rates were 60%, 45%, and 20% in Groups Ia, Ib, and II, respectively, with a statistically significant difference between Groups Ia and II (*p*: 0.01). * Conclusion.* Fiber optic illuminated Ando plombe allows better positioning under the macula and consequently improves the success rate of epimacular buckling in comparison to PPV with internal tamponade in MMHRD.

## 1. Introduction

Since the introduction of vitrectomy as a treatment for retinal detachment caused by macular holes in high myopic eyes by Gonvers and Machemer in 1982 [1], several additional procedures have gained popularity such as intraocular gas tamponade [2], silicone oil tamponade [3], laser photocoagulation to the edge of the hole [4], and internal limiting membrane (ILM) peeling [5], so as to increase the success rate of pars plana vitrectomy (PPV) in these cases.

Epimacular buckling (EMB) in highly myopic eyes has been thought to be technically challenging because of difficulties in placement of buckling material over the macula. So, for some time, vitrectomy was the preferred procedure for this relatively complicated type of detachment [6].

Recently, with the revival of EMB, the correct placement under the hole and the right height for the indentation which alleviates the stretched macular area is still an issue [7].

Bedda introduced the fiber optic guided Ando plombe as a solution for cases with recurrent MHRD at the Vail Vitrectomy meeting March 2013 [8], which allowed accurate positioning under the macula and consequently improves the success rate of the surgery.

The purpose of the present paper is to compare this new modified Ando plombe equipped with a fiber optic light and PPV with gas or silicone tamponade for cases with macular hole retinal detachment (MHRD) in high myopic eyes.

## 2. Patients and Methods

A prospective interventional randomized case series comparing the functional and anatomical outcomes of two surgical procedures for the treatment of MHRD was conducted.

The study included 60 eyes diagnosed with retinal detachment associated with macular hole due to high myopia (axial length > 26 mm). We excluded patients with history or evidence of any ocular trauma; previous intraocular surgery (except for an uncomplicated cataract surgery); age related macular degeneration; proliferative vitreoretinopathy worse than Grade B; inflammatory, vascular, or macular degenerative disease likely to confound visual outcomes; and cases with duration of symptoms of >6 months. All subjects signed a written informed consent after thorough explanation of the procedure and all the procedures were approved by the ophthalmology department institutional review board and the ethics committee of Alexandria Main University Hospital before January 2012.

Patients were randomly assigned to (Group I) PPV with internal tamponade (silicon oil tamponade, Ia, or intravitreal sulfur hexafluoride (SF_6_) gas injection, Ib) or EMB (Group II). The randomization was performed based on computer-generated numbers. Fundus photography, axial A scan ultrasonography, and optical coherence tomography (OCT, Cirrus HD-OCT 4000, version 5.0, Carl Zeiss Meditec) before and after operation were performed whenever possible.

*Surgical Technique*. All surgeries were performed by one single surgeon (AB) between January 2012 and January 2014.

*Vitrectomy with Internal Tamponade*. The standard procedure for MH repair, including a 23-gauge three-port PPV, was performed under general anesthesia in all patients. After core vitrectomy, posterior hyaloid detachment was attempted with the vitrectomy cutter in the suction mode. We looked for residual posterior vitreous cortex by suction after application of triamcinolone acetonide and removed it when present. ILM peeling was not attempted in any case. The peripheral vitreous was removed as much as possible.

Then, the peripheral retina was inspected with sclera depression to detect the presence of any retinal tears. If retinal tears were discovered, laser photocoagulation was applied during the surgery. At the completion of surgery, internal tamponade with silicone oil (viscosity, 1,000 cst; specific gravity, 0.974) or with gas (15%) sulfur hexafluoride (SF_6_) was used. The use of either was according to the random tables previously assigned and not according to a specific case need. The patients were asked to stay prone for 5–7 days. In cases with silicone oil tamponade, removal was scheduled 3-4 months postoperatively.

*Fiber Optic Illuminated Ando Plombe*. The macular buckle used in all cases, also referred to as Ando plombe [6], consists of a T-shaped semirigid silicone rubber rod internally reinforced with titanium wires and an indenting head at one end. The rigid wires permit shaping the explant manually in order to achieve the desired curvature, thus controlling the height of the indentation. We used a length ranging from 25 to 29 mm, chosen according to the globe axial length.

**Figure 1:**
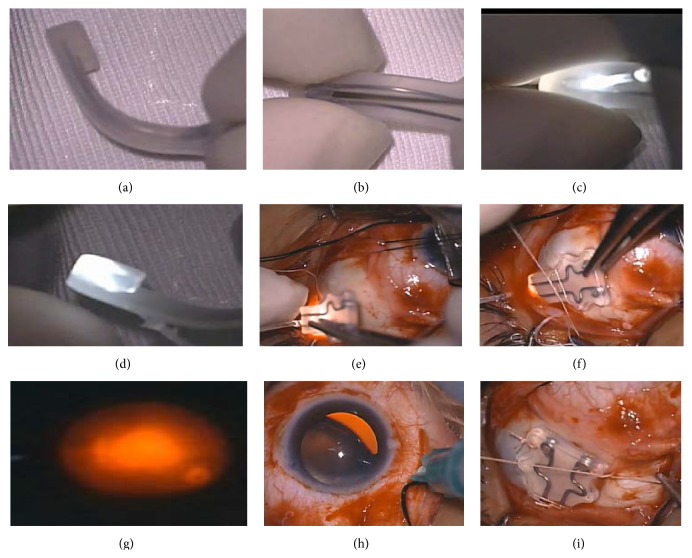
Surgical procedure. (a) Partially bending the Ando plombe to get the desired indentation. (b) A 23-gauge needle inserted towards the indenting head of the explant. (c) A disposable Chandelier fiber (27-gauge) was inserted toward the center of the heel. (d) The terminal knob is illuminated. (e) Placement of the buckle guided with anatomical landmarks. (f) Temporary sutures were taken 18 mm from the limbus between superior and inferior oblique muscle insertions 7 mm apart to keep the Ando plombe from rotating. (g) The glowing head progression could be seen easily owing to the strong illumination of the fiber optic light. (h) Intravitreal air injected to adjust the IOP. (i) The plombe is then secured in place.

Before starting the operation, a 23-gauge needle was inserted towards the indenting head of the explant along the posterior aspect of the buckle to prepare a track. It was then carefully removed and a disposable Chandelier fiber (27-gauge/0.4 mm) (DORC Inc., Netherlands) was inserted toward the center of the heel. The inserted Chandelier optic fiber was fixed to the plombe with two temporary 5-0 nylon sutures (Figure 1).

The surgical procedure included performing 120° superotemporal conjunctival peritomy in order to expose the temporal sclera. Careful dissection of the posterior pole, to identify the posterior edge of the inferior oblique insertion, marks the course of the transverse long posterior ciliary artery (TLPCA). Drainage of the subretinal fluid was performed, to decrease the intraocular pressure (IOP) and height of bullous RD, hence allowing easy identification of the buckle head under the fovea.

While monitoring the fundus using the binocular indirect ophthalmomicroscopy (Oculus BIOM 5, OCULUS Optikgeräte GmbH) system, the glowing head progression could be seen easily owing to the strong illumination of the fiber optic light previously fixed to it.

The plombe's head was adjusted and positioned under the macula. After correct visualization of the indenting head, the tail of the plombe was fixated with additional scleral sutures and the fiber optic light removed. At the end, filtered air was injected to restore normal IOP.

Macular buckling with fibrooptic guided episcleral buckle insertion was the only procedure performed in Group II eyes.

**Figure 2:**
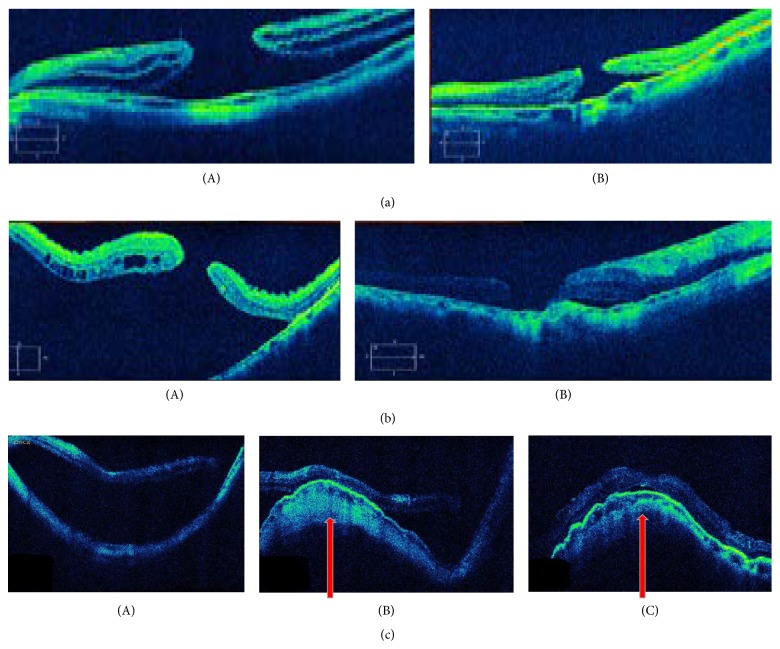
*Upper pictures for OCT* (a). (A) Before fiber optic guided macula buckling. (B) Three weeks after surgery, showing complete reattachment but with persistently opened hole.* Middle OCT cuts* (b). (A) An eye with MHRD three days before the procedure. (B) One week after fiber optic guided EMB showing complete reattachment and reapposition of the hole edge on a horizontal OCT scan.* Lower pictures* (c). (A) OCT shows the subretinal fluid in a vertical cut not passing through the macular hole; (B) shows 1 week after EMB with reapposition of the macula; (C) residual fluid which disappeared 5 days later.

The patients were examined postoperatively on day 1 and week 1 and subsequently at 1, 3, and 6 months and every sixth month thereafter till the end of the follow-up period. The success (final attachment) was estimated regardless of macular hole state (opened or closed) postoperatively; this was estimated after performing the chosen procedure for each group by one month, that is, after the retina became stable from the point of view of the surgeon, and after the gas disappeared completely in Group Ib (Figure 2). The functional outcome was estimated by comparing the visual acuity improvement between the 3 groups in the anatomically successful cases.

All data were collected and assessed by SPSS (SPSS Inc., Chicago, IL, USA) version 20.0 for Windows. For statistical analysis, Snellen visual acuity values were converted to decimal values. The *p* value of 0.05 was regarded as statistically significant.

## 3. Results

The study included 60 patients with 60 eyes classified into two groups: Group I (which was further subdivided into Group Ia, in which PPV and silicone tamponade was done, and Group Ib, in which PPV and gas tamponade was performed) and Group II (which included cases of fiber optic EMB).

**Figure 3:**
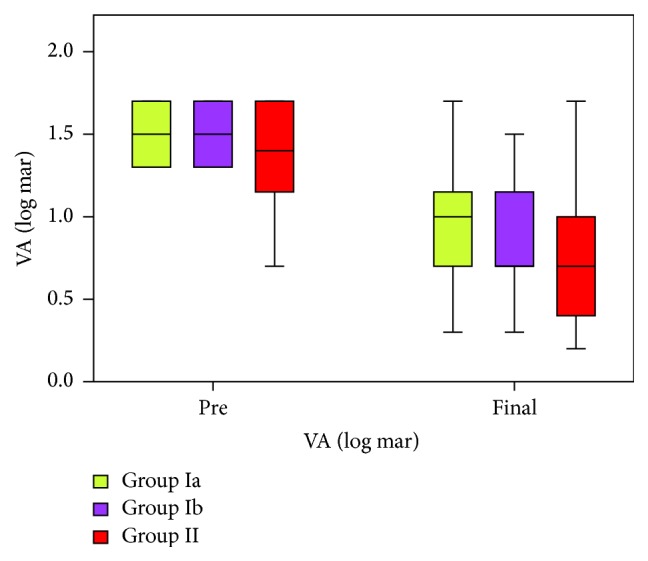
Comparison between the studied groups in relation to preoperative and final visual acuity (VA) in log mar.

Demographic data and the axial lengths of the eyes as measured by A scan US were shown in Table 1. There was no statistically significant difference in axial length before and after the procedure in Groups Ia and Ib. On the other hand, there was a statistically significant difference in axial length in Group II before and after the procedure. This is due to the indentation effect of the macular buckle. The mean VA represented in log mar before the different procedures was 1.51 ± 0.18 in Group Ia, 1.50 ± 0.17 in Group Ib, and 1.35 ± 0.31 in Group II, while the final VA—after 1 month from the procedure—for each group in log mar was 0.95 ± 0.37, 0.88 ± 0.37, and 0.71 ± 0.41 in the 3 groups, respectively (Figure 3).

The change (improvement) in VA was statistically significant in the 3 groups after the procedure. The VA improved by 37.31% in Group Ia, 40.67% in Group Ib, and 49.40% in Group II. Nevertheless, there was no statistically significant difference between the groups after the procedure as regards visual acuity improvement in the anatomically successful cases (*p* = 0.148).

The three groups were followed up till (mean ± SD) 16.50 ± 5.55 months in Group Ia, 18.05 ± 5.24 months in Group Ib, and 15.80 ± 4.88 months in Group II, with the shortest follow-up period in Group II and the longest in Group Ib. This was not statistically significant (*p* = 0.371).

Regarding the anatomical success rate (final attachment), in Group Ia, the attached eyes were 11 out of 20 at the end of the study with total success rate of 55%; the 9 detached eyes were divided into early failure (before removal of silicone) which included 3 eyes and late failure (after removal of silicone) which included 6 eyes. In Group Ib, the attached eyes were 12 out of 20, representing a success rate of 60% at the end of the study, while in Group II the attached eyes were 20 out of 20 with a success rate of 100% at the end of the study. There was a statistically significant difference in the success rate between Group I and Group II at the end of the follow-up period (*p* < 0.001 in Ia, *p*: 0.002 in Ib) (Table 1).

The type of closure of the macular hole in the successful cases in the 3 groups as identified by the postoperative OCT was divided into type 1 closure, in which reapposition of the edges with a central foveolar depression was seen on OCT, and type 2 closure, in which a defect was still identified by OCT but with flat edges. The percentage of type 1 closure in Groups Ia, Ib, and II was 43.75% (7 out of 16 successful cases), 43.75% (7 out of 16 attached cases), and 40% (8 out of 20 successful cases), respectively. The difference between the three groups in the closure rate was not statistically significant as the *p* value was 0.965167.

The incidence of complications rates in Group Ia was the highest. It reached 60% in Group Ia, 45% in Group Ib, and 20% in Group II. There was a statistically significant difference in rate of complications between Group Ia and Group II (*p*: 0.01), but not between Group Ib and Group II (*p*: 0.09).* In Group Ia*, 9 eyes developed cataract during the follow-up period and 4 cases had an elevated IOP, but only 2 were complicated with optic disc damage. In 2 cases, silicone oil adversely entered the AC and was managed by placing the patient in a face-down position for 3 to 5 days. Another case suffered from pupillary block glaucoma which was solved by YAG laser iridotomy.

*In Group Ib*, 6 cases (30%) developed cataract at the end of the follow-up period and 2 cases (10%) had increased IOP at the early postoperative period controlled medically with one antiglaucoma medication, while, in Group II, 3 eyes (15%) had perforation during drainage of subretina fluid and one case (5%) was complicated with buckle migration 10 months from surgery which was successfully repositioned later; despite this complication, MH remained closed (Table 2).

## 4. Discussion

The new modified fiber optic illuminated EMB procedure has the advantage described by Ando et al. [6] of safe placement on the equatorial episclera without extraocular muscles disinsertion, reducing the potential damage of nerves and vessels around the optic nerve. Also, it has the advantage of accurate placement under the macular hole, with a sufficient indentation height to reduce the axial length of the eye which changes the shape of the posterior pole from being extremely concave because of the posterior staphyloma to having a convex configuration. This will relieve both tangential and anteroposterior traction over the posterior pole. Moreover, macular buckling avoids cataract progression and iatrogenic breaks, which are common risks of vitrectomy [9].

In the current case series, permanent reattachment of the retina was achieved in 20 (100%) of 20 eyes after single EMB surgery without any remarkable intraoperative or postoperative complications. In the vitrectomy group, the success rate was 55% in the silicone tamponade group (Ia) and 60% in the SF_6_ tamponade group (Ib). These anatomical results for the fiber optic illuminated EMB are better than most previous reports of traditional EMB [6, 10, 11]. Again, the anatomical outcomes after fiber optic guided EMB were better than those after PPV.

The change (improvement) in VA was statistically significant in each of the 3 groups after the procedure but with no statistically significant difference between the groups in the anatomically successful cases (*p* = 0.148). This means that, regardless of the procedure used to reattach the retina in cases with MHRD, there will be a significant improvement in the visual acuity of the patient, with no advantage of a specific procedure over the other. To our knowledge, no previous work compared the sole use of the fiber optic EMB with vitrectomy in cases of MHRD in high myopic eyes.

The macular hole closure rate was 43.75% in Group I (Ia or Ib) and 40% in Group II; this was statistically insignificant, *p* = 0.965. One would expect the closure rate in the vitrectomy group to be higher, but again ILM peeling was not attempted in any case for fear of causing iatrogenic breaks in such thinned out areas of the retina.

The reason of closure of some macular holes in the current study in the macular buckle group was assumed to be due to the change in the configuration of the posterior pole to a convex shape together with opposing the countertraction exerted by the posterior staphyloma in that part, consequently approximating the edges of the hole allowing some to actually close. Alkabes et al. [10] achieved a higher closure rate in primary myopic macular hole detachment cases of 81% (17 out of 21 cases) and 57% (12 out of 21 cases) closure rate in recurrent cases. They performed macular buckling and PPV in all patients.

Ando et al. [6] reported in their EMB group a success rate of 93.3% after primary surgery and 100% after secondary surgery. Again, in the PPV group, the retinal reattachment rate was comparable to our results in Group I, reaching 50% after primary surgery and 86% after secondary surgery using the EMB procedure.

Later on, different studies used the Ando plombe like in the work of Sasoh et al. [11], who in 33 cases of MHRD achieved an anatomical success rate of 94% after primary EMB and 100% after secondary EMB. Ripandelli et al. [12] performed the surgery using solid silicone exoplant sutured into the sclera 5 mm above and below the macular hole; these investigators achieved retinal reattachment in 14 (93%) of 15 highly myopic eyes with MHRD after primary surgery. Stirpe et al. [13] introduced adjustable macular buckling device characterized by the possibility of adjusting the amount and localization of indentation, but they did not conduct a head-to-head study with PPV; in fact, they still acknowledged PPV as the primary procedure for most staphyloma associated conditions.

Siam et al. [9, 14] reported, through a new insight regarding the anatomical topography of the posterior pole of the globe, a buckling technique with 100% success rate. But their surgical technique involved severing the superior oblique as well as placing two posterior sutures very close to the optic nerve without damaging posterior ciliary vessels. Despite this somewhat difficult technique, one case still showed malposition of the buckle postoperatively.

Other studies reached a success rate comparable to our result (100% success rate), but they utilized macular explant together with performing a PPV. A study conducted by Mortada [15] evaluated a novel technique of episcleral macular buckling in 15 highly myopic eyes (axial length > 30 mm) with postvitrectomy recurrent macular hole retinal detachment. A 7 mm silicone sponge strengthened with a U-shaped, 0.5 mm orthodontics stainless steel wire fed along its length and hand-bent to produce an L-shaped buckle was used. Successful retinal reattachment, with improvement in visual acuity, was achieved in all 15 eyes. Closure of the macular holes was confirmed by OCT.

Mateo et al. [7] utilized their surgical technique in 4 patients with high myopia and different underlying pathologies in which they inserted an optical fiber coupled to an Ando plombe; this was done concomitantly to performing pars plana vitrectomy with posterior hyaloid dissection, as well as ILM peeling after staining with Membrane Blue. Resolution of the macular pathology occurred in all cases. The small number of cases (only 4), performing the EMB with vitrectomy and ILM peeling, makes it difficult to assess the value of the illuminated buckle in cases with MHRD.

The current study showed complication rate of 20%. This was acceptable in view of the technical ease and high success rate. Other studies like Siam et al. [9] reached a complication rate of 65%. The rate of perforation (15%) in Group II seems apparently higher than the other two groups; this may be explained by the fact that this occurred in the earlier cases enrolled in the study before the surgeon realized the extremely thin sclera which the sutures have to be put in. In the later cases extreme caution and meticulous technique prevented this complication from happening.

In conclusion, macular buckling with fiber optic guided Ando plombe offers an effective solution when considering both the anatomic and the functional perspective for repair of cases of MHRD when compared to PPV with internal tamponade.

Illumination of the terminal button of the Ando plombe allows better positioning under the macula and consequently improves the success rate of the surgery. Combining both PPV with illuminated Ando plombe might give the best chances of anatomical success as well as visual improvement.

## Figures and Tables

**Table 1:** Demographic data of the studied groups.

	Group Ia		Group Ib		Group II		Test of sig.	*p* value
	(*n* = 20)		(*n* = 20)		(*n* = 20)			
	Number	%	Number	%	Number	%		
Sex								
Male	3	15.0	7	35.0	5	25.0	*χ* ^2^ = 2.133	0.344
Female	17	85.0	13	65.0	15	75.0		
Eye								
OD	11	55.0	10	50.0	10	50.0	*χ* ^2^ = 0.133	0.935
OS	9	45.0	10	50.0	10	50.0		
Age								
Min–max	35.0–65.0		35.0–65.0		35.0–70.0		*F* = 1.239	0.297
Mean ± SD	47.85 ± 8.09		50.50 ± 9.43		52.5 ± 10.44			
Median	47.50		49.50		51.50			
Axial length								
Before								
Min–max	27.0–34.0		27.0–34.50		27.0–34.0		0.436	0.297
Mean ± SD	30.03 ± 2.03		30.17 ± 2.25		30.63 ± 2.06			
Median	30.0		29.75		30.75			
After								
Min–max	27.5–34.5		27.0–35.0		26.0–35.5		2.078	0.135
Mean ± SD	30.28 ± 1.75		30.35 ± 2.46		29.23 ± 1.53			
Median	30.0		29.75		29.75			
*p*_1_	0.204		0.420		<0.001^*∗*^			
Success rate								
After procedure						
Attached	11	55.0	12	60.0	20	100.0
Detached	9	45.0	8	40.0	0	0.0
*p*_2_	11.61^*∗*^ (<0.001^*∗*^)		10^*∗*^ (0.002^*∗*^)					

*χ* ^2^: Chi square test.

*F*: *F* test (ANOVA).

*p*_1_: *p* value for paired *t*-test for comparing between “before” and “after” in each group in relation to axial length.

*p*_2_: *p* value for comparison of each group with Group II.

^*∗*^Statistically significant at *p* ≤ 0.05.

**Table 2:** Complications occurring in each group during the study period.

	Group Ia		Group Ib		Group II	
	(*n* = 20)		(*n* = 20)		(*n* = 20)	
	Number	%	Number	%	Number	%
Complication						
No	8	40.0	11	55.0	16	80.0
Yes	**12**	**60.0**	**9**	**45.0**	**4**	**20.0**
*χ* ^2^_1^*∗*^_ (*p*)	6.6667 (0.01)					
*χ* ^2^_2_ (*p*)	2.849 (0.09)					
*χ* ^2^_3_ (*π*)	0.9023 (0.34)					
Perforation	0	0.0	0	0.0	3	15.0
Migration of buckle	0	0.0	0	0.0	1	5.0
Cataract	9	45.0	6	30.0	0	0.0
Increased IOP	2	10.0	2	10.0	0	0.0
Glaucoma	2	10.0	0	0.0	0	0.0
Expulsive hemorrhage	1	5.0	0	0.0	0	0.0
Silicon in AC	2	10.0	0	0.0	0	0.0
Pupillary block	1	5.0	0	0.0	0	0.0
Vitreous hemorrhage	0	0.0	1	5.0	0	0.0

*χ* ^2^_1_: value for Chi square for comparing between Group Ia and Group II.

*χ* ^2^_2_: value for Chi square for comparing between Group Ib and Group II.

*χ* ^2^_3_: value for Chi square for comparing between Group Ia and Group Ib.

^*∗*^Statistically significant at *p* ≤ 0.05.
